# Effect of Glucose Concentration on Electrochemical Corrosion Behavior of Pure Titanium TA2 in Hanks’ Simulated Body Fluid

**DOI:** 10.3390/ma9110874

**Published:** 2016-10-26

**Authors:** Shuyue Liu, Bing Wang, Peirong Zhang

**Affiliations:** 1 High School Attached to Shandong Normal University, Jinan 250014, China; liushuyue2000@gmail.com; 2 Key Laboratory of High Efficiency and Clean Mechanical Manufacture of MOE, School of Mechanical Engineering, Shandong University, Jinan 250061, China; sduzhangpeirong@gmail.com; 3 Department of Mechanical Engineering, Michigan State University, Lansing, MI 48910, USA

**Keywords:** glucose concentration, corrosion, pure titanium, simulated body fluid, implant

## Abstract

Titanium and its alloys have been widely used as implant materials due to their excellent mechanical property and biocompatibility. In the present study, the effect of glucose concentration on corrosion behavior of pure titanium TA2 in Hanks’ simulated body fluid is investigated by the electrochemical impedance spectrum (EIS) and potentiodynamic polarization methods. The range of glucose concentrations investigated in this research includes 5 mmol/L (limosis for healthy people), 7 mmol/L (after diet for healthy people), 10 mmol/L (limosis for hyperglycemia patient), and 12 mmol/L (after diet for hyperglycemia patient), as well as, 15 mmol/L and 20 mmol/L, which represent different body fluid environments. The results indicate that the pure titanium TA2 demonstrates the best corrosion resistance when the glucose concentration is less than 10 mmol/L, which shows that the pure titanium TA2 as implant material can play an effective role in the body fluids with normal and slight high glucose concentrations. Comparatively, the corrosion for the pure titanium implant is more probable when the glucose concentration is over 10 mmol/L due to the premature penetration through passive film on the material surface. Corrosion defects of pitting and crevice exist on the corroded surface, and the depth of corrosion is limited to three microns with a low corrosion rate. The oxidation film on the surface of pure titanium TA2 has a protective effect on the corrosion behavior of the implant inner material. The corrosion behavior of pure titanium TA2 will happen easily once the passive film has been penetrated through. The corrosion rate for TA2 implant will accelerate quickly and a pure titanium implant cannot be used.

## 1. Introduction

Titanium and its alloys have excellent properties such as high specific strength (i.e., strength-to-weight ratio, etc.), high toughness, high corrosion resistance and excellent biocompatibility [1,2,3]. They have been widely used as implant materials in such occasions as dental implant [4], tooth orthopedic line [5], artificial joint [6], bone trauma product [7], interventional cardiovascular stent [8], etc. The titanium alloy Ti6Al4V has once been applied as the main implant material in the biomedical field, due to its enhanced mechanical properties with the addition of such alloy elements as aluminum and vanadium. However, the dissolution of poisonous elements has been noticed for implants made of Ti6Al4V recently, which leads to some negative effects related to human physiological health [9,10]. Comparatively, the application of pure titanium implants is much safer which can reduce the secondary hazards of implant materials on human physiological health [11].

Pure titanium has very high chemical activity, so it is apt to be passivated by oxygen within the atmosphere of air or oxygen-rich solutions. Pure titanium is usually regarded as self-passivating metal. There is a layer of compact oxidation film formed on the material surface of pure titanium after self-passivation and its thickness ranges from several nanometers to tens of nanometers. Consequently, the pure titanium owns excellent corrosion resistance. Previous research has revealed that the oxidation film on pure titanium is in a state of dynamic balance between dissolution and repair (i.e., second passivation) processes within solutions [12]. If the dynamic balance state is destroyed by some specific solution components or other physical and chemical factors, the corrosion degree of pure titanium may be aggravated dramatically. 

Surface corrosion is the main problem that the metal implant materials meet in clinical application. The main type of corrosion for biomedical metals is localized corrosion [13]. It means that if one localized area is corroded, the rest area of the surface is nearly undamaged. The existence of microorganism or some specific chemical components may not destroy the implant surface directly, but they can create the necessary conditions for electrochemical corrosion leading to severe damage on implant materials.

Over one thousand tons of titanium and titanium alloys are used as implant materials in the world every year [14]. Although the corrosion resistance of these kinds of materials is much better that the other metals, their corrosion problem especially for receptors with special corporeity, has still attracted much attention gradually in recent years. For example, precious research [15,16] has found severe health problems caused by the bone anchored hearing aid made of titanium alloys. They found that there were titanium particles spreading around the implant hearing aid within the soft tissue of receptors, which leaded to the localized inflammation. The titanium particles were not residual fragments produced during surgery process. They were also not caused by wear because ears are not movable organ. The main reason leading to the formation of titanium particles was the corrosion of implant material surface, which subsequently caused the fragments separation from the implant surface. Rogers et al. [17] carried out research to determine whether in vitro studies would detect differences in the cellular response to wear particles of titanium alloy and pure titanium commonly used in the manufacture of joint replacement prostheses. They researched the particles with the order of 1 micron in diameter representative of those found adjacent to failed prostheses. Exposure of human monocytes to Ti6Al4V at concentrations of 4 × 10^7^ particles/mL produced a mean prostaglandin E2 release of 2627.6 pM, while commercially-pure titanium particles induced a release of only 347.8 pM. In addition, Ti6Al4V stimulated significantly more release of the other cell mediators, interleukin-1, tumor necrosis factor and interleukin-6. Their results showed that the pure titanium is recommended to be used as implant material instead of titanium alloys in order to avoid negative effects on human health.

This paper aims to research the corrosion behavior of pure titanium TA2 used as implant material within the atmosphere of Hanks’ simulated body fluid (SBF). In addition, the body fluids of hyperglycemia patients with special corporeity are selected as the research objective. The difference of corrosion behavior of implant material TA2 used in the simulated body fluids of healthy people and hyperglycemia patients will be compared. The electrochemistry method is adopted to investigate the corrosion behavior of TA2 implants within different Hanks’ SBFs. The polarization curve and corrosion potential variation are researched to assess the corrosion resistance of the implant material. The research will reveal the effect of blood glucose concentration on the corrosion behavior of TA2 implant material, which can help to lay a solid theoretical foundation for improvement of the corrosion resistance of pure titanium implants. The research results can also help to instruct the clinical application of pure titanium implants in surgery.

## 2. Material and Experimental Procedure

### 2.1. Experimental Material and Electrode Preparation

Pure titanium TA2 was selected as the research objective which has excellent mechanical properties and good corrosion resistance. The chemical compositions (wt.%) of TA2 include Fe ≤ 0.30, C ≤ 0.10, N ≤ 0.05, H ≤ 0.015, O ≤ 0.25, and the rest is Ti. Pure titanium TA2 used in this research was purchased from Baoji Titanium Industry Company Limited (Baoji, China). The cylindrical specimens with dimensions of *Φ*6 mm × 2 mm were prepared through the procedures of wire electrical discharge machining and polishing.

The back surfaces of specimens were connected with copper interconnects through welding technique or conductive adhesive. After detection of connecting reliability with multimeter, the specimens were packaged with epoxy resin. E-51 epoxy resin was used as the casting material. Methylhexahydrophthalic anhydride (M-HHPA), N,N-Dimethyl benzyl amine (BDMA) and fumed silica (SiO_2_) were selected as the curing agent, the curing accelerator and the padding, respectively. The above materials were mixed evenly with the weight ratio (wt.%) of E-51:M-HHPA:SiO_2_:BDMA = 400:240:150:1. Then the mixed material was degassed in the vacuum drying oven under 40 °C, after that the mixed material was filled into a specifically designed die to make the electrodes. At last, the casting epoxy resin TA2 electrodes were placed into the vacuum drying oven for solidification. The solidification process was carried out under 80 °C for 2 h combined with 120 °C for 4 h. After the procedure of solidification, the epoxy resin TA2 electrodes were air-cooled to room temperature.

The TA2 electrodes were grinded with raw emery paper to make sure that the front surfaces of electrodes were uncovering. Then they were further grinded using abrasive papers with particle size of 400#, 800#, 1200# and 2000#. The grinded electrodes were polished with lint and ultrasonic cleaned with acetone and absolute ethyl alcohol sequentially. The impurities including grease on the specimen surfaces were wiped off and then the specimen surfaces were dried with nitrogen. The ultimate surface roughness *R_a_* of the specimens prepared for the electrochemical test is 0.2 μm.

### 2.2. Experimental Setup of Electrochemical Corrosion

Table 1 shows the ranges of blood glucose concentration for healthy people and hyperglycemia patient. Because the body fluid environment is complex and changeful, the blood glucose concentration varies at different time or before and after the diet, which will directly affect the corrosion behavior of titanium implants. The variations of corrosion behavior for titanium implants determine their service lives and even do harm to the body health and life safety of patients. Based on the information in Table 1, the range of blood glucose concentration investigated in this research is then determined for healthy people and hyperglycemia patient, The background of diets for the variation of glucose concentrations in Table 1 is daily normal diets [18].

The experiments of electrochemical corrosion for TA2 were carried out on CHI 604A electrochemical workstation produced by Shanghai Chenhua Instrument Co., Ltd. (Shanghai, China). The other experimental instruments used are introduced in Table 2. The electrolytic tank of three electrodes system was adopted in this research, during which the TA2 electrode was the working electrode. The saturated calomel electrode and platinum gauze electrode with the cross-sectional area of 1 cm^2^ were selected as the reference electrode and auxiliary electrode, respectively.

The etchant solutions included analytical reagents and Hanks’ SBFs. The Hanks’ SBFs were prepared using deionized water. The components of SBFs include NaCl 8 g + KCl 0.4 g + CaCl_2_ 0.14 g + NaHCO_3_ 0.35 g + MgCl_2_·H_2_O 0.1 g + MgSO_4_·H_2_O 0.06 g + KH_2_PO_4_ 0.06 g + Na_2_HPO_4_·H_2_O 0.06 g. All these solutes were dissolved in deionized water and diluted to 1000 mL, after that the Hanks’ SBFs were prepared well. There were six groups of Hanks’ SBFs in total prepared for the experiments, and there were five specimens tested in every group of Hanks’ SBFs. The glucose C_6_H_6_O_6_ with weights of 0.9 g, 1.26 g, 1.8 g, 2.16 g, 2.7 g and 3.6 g per 1000 mL was added into the six groups of Hanks’ SBFs. Consequently, the SBFs with different glucose concentrations, 5 mmol/L (limosis for healthy people), 7 mmol/L (after diet for healthy people), 10 mmol/L (limosis for hyperglycemia patient), 12 mmol/L (after diet for hyperglycemia patient), 15 mmol/L and 20 mmol/L, respectively, were obtained. The analytic reagents used in the experiments are presented in Table 3.

The three electrodes, the working electrode, the reference electrode and the auxiliary electrode were immersed into different SBFs with varied glucose concentrations for 3000 s, which could then form the stable electrochemical systems and the open circuit potential (OCP) was measured.

The measurement of electrochemical impedance spectroscopy (EIS) was carried out under the condition of OCP. The frequency range of measurement was set as from 10^–2^ Hz to 10^5^ Hz and the amplitude of sine altering current was set as 5 mV. The EIS data were analyzed using the software of Zsimp-win.

During measurement of the potentiodynamic polarization curve, the scanning range of the electric potential was set as ±200 mV with respect to the OCP, and the scanning speed was set as 10 mV/s. The Tafel curve was fitted base on the extrapolation method, through which the electrochemical corrosion parameters including the self-corrosion potential and the self-corrosion current were determined.

## 3. Results and Discussion

### 3.1. Measurement and Analysis of EIS

**Figure 1:**
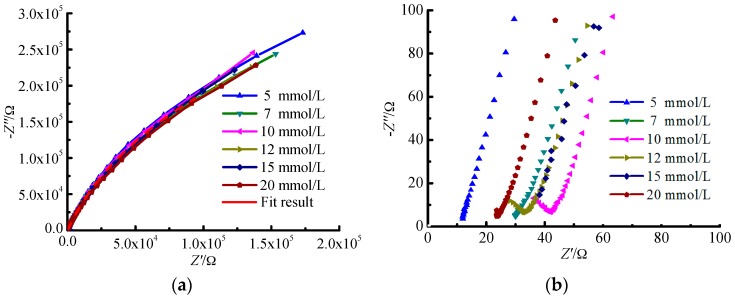
Nyquist figure and enlarged view of high frequency area for TA2 under different glucose concentrations. (**a**) Nyquist figure; and (**b**) enlarged view of high frequency area.

**Figure 2:**
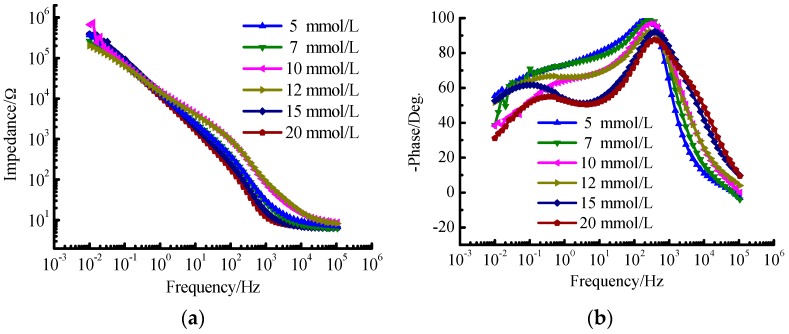
Bode figure for TA2 under different glucose concentrations. (**a**) lg|*Z*| versus lg*f* curve; and (**b**) −*θ* versus lg*f* curve.

The results of EIS for TA2 under different glucose concentrations are shown in Figure 1 and Figure 2. It can be seen from Figure 1a that the Nyquist curves of TA2 under different glucose concentrations can be all regarded as one part of arcs. The Nyquist curves corresponding to low frequency area belong to arcs with large capacitive reactance as shown in Figure 1a, while those corresponding to high frequency area belong to arcs with small capacitive reactance as shown in Figure 1b. Thus, there are two time constants to be determined. When the frequency ranges from 0.01 Hz to 1 Hz, the polarization resistance of TA2 specimen exceeds 10^5^ Ω·cm^2^ as shown in Figure 2a, and the phase angle approaches −60° as shown in Figure 2b. 

The arc in Nyquist figure reflects the impedance magnitude during the electron transfer process on electrode surface. The bigger the arc in Nyquist figure is, the larger resistance for electron transfer will be. The larger resistance for electron transfer means that the capture and loss of electrons are difficult, i.e., the metal is difficult to be dissolved or corroded.

When the glucose concentration ranges from 10 mmol/L to 12 mmol/L, the reverse capacitive reactance arc occurs within the high frequency area in the EIC figure as shown in Figure 1b. The reason is that one layer of steady passivated film is formed on the specimen surface after a period of reaction time between the specimen and the Hanks’ SBF, which improves the corrosion resistance of TA2 specimen. The passivated layer equals to a shielding layer with large resistance and low capacitance. It isolates the further contact between the specimen inner material and the solution that protects the specimen inner material from being corroded. It can be seen that there are significant variations for the phase profiles in Figure 2b especially for the glucose concentratios of 15 mmol/L and 20 mmol/L. The reason is that the aggregation of glucose on the specimen surface increases with the increase of glucose concentration, which enhances the phenomenon of double-layer capacitor and induces the double peak phenomenon. Actually, the tendency for the formation of this phenomenon has been presented under the glucose concentration of 10 mmol/L.

**Figure 3:**
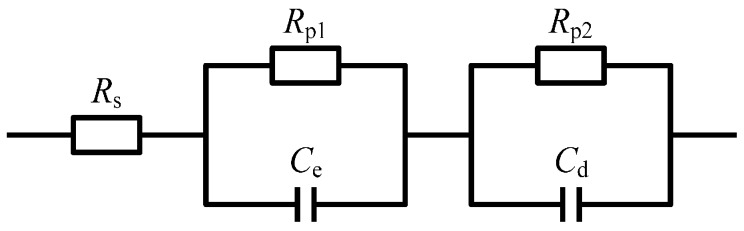
Equivalent circuit diagram for TA2 within.

From the Bode phase angle plot (Figure 2b), at least two time constants are required to fit the EIS results. The oxidation film formed on TA2 specimen consists of compact inner oxidation layer and loosened outer oxidation layer. The inner and out oxidation layers have different electrochemical kinetics characteristics. Figure 3 presents the equivalent circuit diagram as *R*_s_(*R*_p1_*C*_e_)(*R*_p2_*C*_d_) for TA2 within the Hanks’ SBF, where *R*_s_ denotes the solution resistance of the electrode system. *R*_p1_ and *R*_p2_ denote the values of resistance for the inner oxidation layer and the outer oxidation layer, respectively; and *C*_e_ and *C*_d_ denote the values of capacitance for the inner oxidation layer and the outer oxidation layer, respectively.

According to the equivalent circuit diagram in Figure 3, the EIS data were fitted using the software of Zsimp-win. Then the dependence of electrochemical corrosion properties of TA2 electrode on the glucose concentration can be determined as shown in Table 4. The constant phase elements (CPEs) instead of pure capacitances are applied to improve the fitting precision due to the distribution of relaxation times resulting from inhomogeneity at the electrode surface at nano/micro scale (e.g., roughness, porosity, etc.). The electrochemical corrosion properties include the resistance and capacitance for the two-layer oxidation film structure on specimen surface. Comparatively, the resistance of compact inner oxidation layer is larger leading to better corrosion resistance, while the resistance of loosened outer oxidation layer is less leading to inferior corrosion resistance. The reason lies in that the absorbability of loosened oxidation layer is more powerful, so the etchant solution absorbs into the loose pores of the oxidation layer causing the aggravation of corrosion.

### 3.2. Measurement and Analysis of Potentiodynamic Polarization Curve

**Figure 4:**
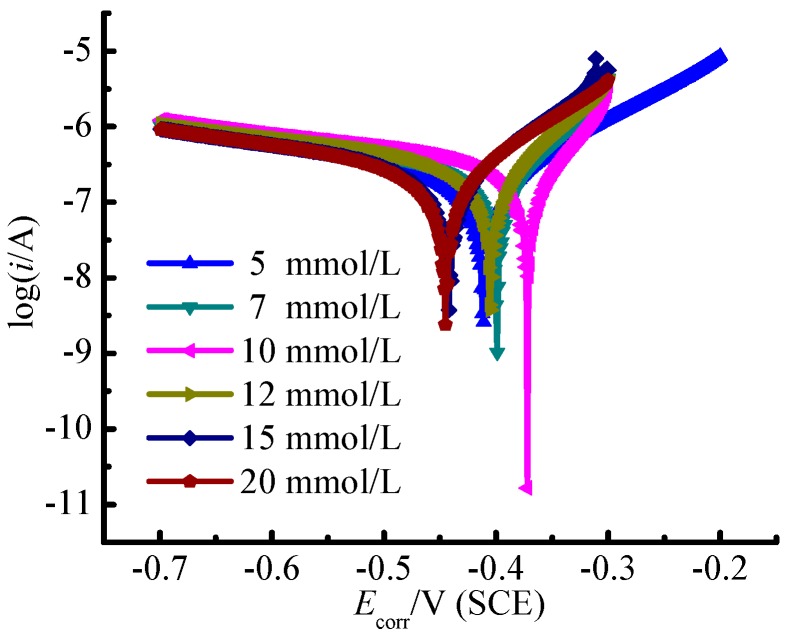
Potentiodynamic polarization curves of TA2 in different SBFs with varied glucose concentrations.

Figure 4 presents the potentiodynamic polarization curves of the test material TA2 in different SBFs with varied glucose concentrations. The electrochemical corrosion parameters, such as the corrosion potential (*E*_corr_), the corrosion current (*I*_corr_), the polarization resistance (*R*_p_) as well as the anodic and cathodic Tafel slopes (*β_a_* and *β_c_*, respectively) determined by Tafel curves are summarized in Table 5.

It can be seen in Figure 4 that the implant material TA2 presents stimulated corrosion state under all investigated varied glucose concentrations. With the corrosion potential increasing, the corrosion current also enhances. With the glucose concentration in Hanks’ SBF increasing, the Tafel curve first shifts to the right (i.e., more passivating status) and then shifts to the left. The results indicate that the corrosion resistance of TA2 is the strongest under the glucose concentration of 10 mmol/L. When the corrosion voltage exceeds 0.5 V, the Tafel curves present the condition of passivating and the corrosion currents tend to be steady. It demonstrates that the deterioration and the formation of passivating film on TA2 specimen surface have reached a balance that is independent on the glucose concentration. According to the fitting results of polarization curves in Table 5, the corrosion potential of the specimen in the environment of glucose concentration with 10 mmol/L is the highest, which means that TA2 is the most difficult to be corroded under this glucose concentration. However, the specimen surface is very easy to be corroded once the oxidation film is damaged because the polarization resistance will be very small in this situation. It is attributed to that anions are prone to be adhesion upon the surface due to the promotion effect of glucose concentration, with which the passive film will be easily penetrated through, then the corrosion will happen.

### 3.3. Surface Observation

**Figure 5:**
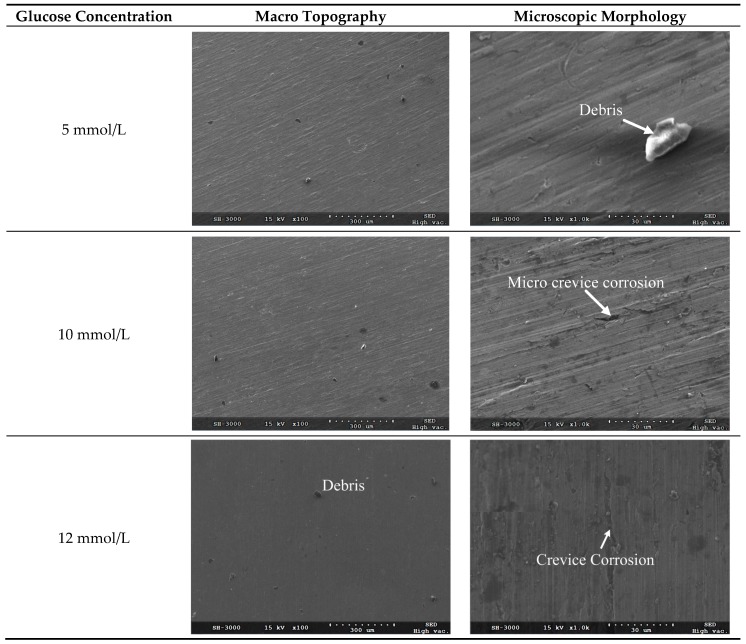

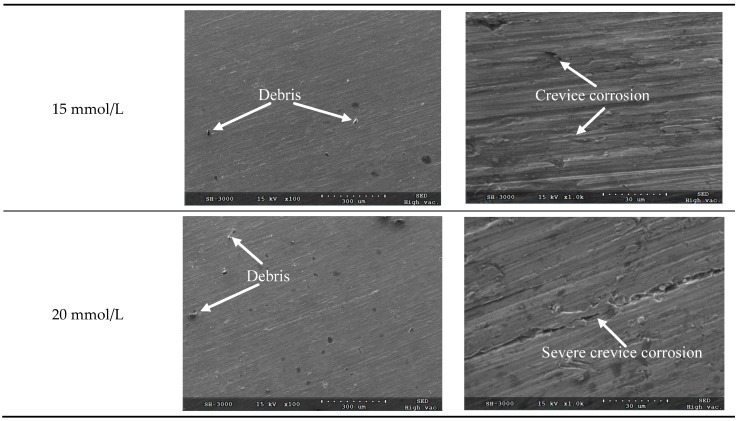
SEM micrographs for the corroded surface of TA2 electrode under different glucose concentrations.

After removal of corrosion products and residual chemicals using ultrasonic cleaning with anhydrous ethanol, the scanning electron microscope (SEM) micrographs for the corrosion surface of TA2 electrode under different glucose concentrations were obtained as shown in Figure 5. Figure 5 shows the macro topography and microscopic morphology for the corrosion surfaces under the glucose concentrations of 5 mmol/L, 10 mmol/L, 12 mmol/L, 15 mmol/L and 20 mmol/L, respectively. It can be seen from Figure 5 that no pitting occurs on the corrosion surface under all investigated glucose concentrations, while a majority of area on the corrosion surface remains passivated state.

It can be seen from the microscopic morphology in Figure 5 that there are slight crevices observed on the corroded surface when the glucose concentrations are less than 20 mmol/L. Because there are chloridions (from NaCl, MgCl_2_ and KCl) in the Hanks’ SBF, the metal ions are apt to bind with the chloridions which leads to the surface corrosion. It demonstrates that the corrosion belongs to localized corrosion pattern and the corrosion rate is very low.

**Figure 6:**
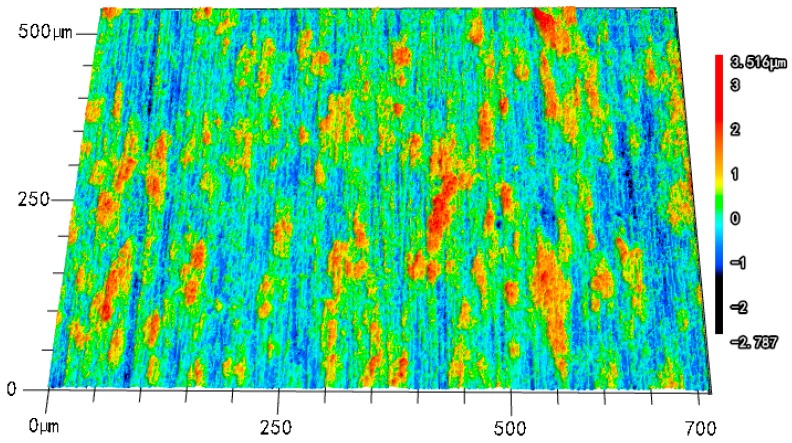
Three dimensional micrograph for the corroded surface of TA2 electrode under glucose concentration of 12 mmol/L.

Figure 6 presents the three dimensional micrographs for the corroded surface of TA2 electrode obtained under the glucose concentration of 12 mmol/L. It can be seen that the corrosion rate of TA2 specimen in the Hanks’ SBF is very low and the corrosion depth on the surface is less than three micrometers. During the corrosion period of TA2 specimen, the effect of tiny corrosion on the implant can be neglected. However, when the metal irons dissolved into the body of receptor accumulate for a long time, they will do harm to the somatic function of the receptor which will cause inflammation or even endanger the life of the receptor [19,20].

The important information for corrosion behavior of TA2 implant material in Hanks’ SBF can be obtained based on the Tafel extrapolation method and the EIS measurement. With the increase of blood glucose concentration, the corrosion resistance of TA2 specimen first increases and then decreases. When the glucose concentration is 10 mmol/L, the corrosion resistance of TA2 specimen is the strongest. The research results have demonstrated that the pure titanium TA2 can be used as the implant material. Based on the lab experimental results, when the blood glucose concentration is normal or a bit higher than the normal value, the implant material of TA2 can maintain excellent corrosion resistance for a long time. The TA2 implant can be used to help receptor with no hyperglycemia to recover somatic function, but the receptor should keep blood glucose concentration in balance during the use of TA2 implant. Because the research results are obtained using lab experiments, the in vivo behavior of TA2 as implant material and its corrosion behavior in real body fluid should be re-examined.

However, for the hyperglycemia patients whose blood glucose concentration is much higher than the normal value, the corrosion rate and corrosion degree of TA2 implant will probably be much larger compared with those of normal receptors. This may cause the damage of pure titanium implant and lead to second damage to receptors. 

## 4. Conclusions

This paper has investigated the corrosion behavior of pure titanium TA2 in Hanks’ SBF, through which the effect of blood glucose concentration on the surface corrosion for pure titanium TA2 has been revealed. The research results can provide theoretical foundation for clinical application of titanium implant. The research found that the pure titanium TA2 presented the best corrosion resistance within the glucose concentration of 10 mmol/L. It demonstrates that TA2 titanium can be used as the implant material at normal blood glucose concentration and a bit higher blood glucose concentration. The corrosion defects of crevice were observed on the TA2 material surface, but the corrosion rate for TA2 in Hanks’ SBF was very low. The oxidation film formed on the TA2 surface can protect the inner material from being corroded during initial corrosion stage. The corrosion behavior of pure titanium TA2 will happen easily once the passive film has been penetrated through. As a result, the implant material should avoid to be rubbed during surgery operation. When the blood glucose concentration is higher than 12 mmol/L for hyperglycemia patients, the corrosion rate for TA2 implant will accelerate quickly and the pure titanium implant cannot be used then. Thus, it should be cautioned for hyperglycemia patients to use such pure titanium implants. Research is also expected to provide technical supports to the surgery clinical application of titanium implants.

## Figures and Tables

**Table 1:** The range of blood glucose concentration for healthy people and hyperglycemia patient [18].

Research Object	Body Environment	Blood Glucose Concentration/mmol·L^−1^
Healthy people	Limosis	4.4–5.6
	Two hours after diet	≤7.8
Hyperglycemia patient	Limosis	>7.0
	Two hours after diet	≥11.1

**Table 2:** Experimental instruments.

Instrument Name	Specification	Manufacturer
Electrochemical workstation	CHI 604A	Shanghai Chenhua Instrument Co., Ltd. (Shanghai, China).
Vacuum drying oven	ZKXF-1	Shanghai Shuli Instrument Co., Ltd. (Shanghai, China).
Deionized water equipment	UPT-I-107	Chengdu Chaochun Science and Technology Co., Ltd. (Chengdu, China).
Electronic scales	AUY 120	Shimadzu Corporation (Shimane, Japan).
Working electrode	TA2	Self-manufactured (Jinan, China).
Reference electrode	Saturated calomel electrode	Shanghai Yidian Science Instrument Co., Ltd. (Shanghai, China).
Auxiliary electrode	Platinum gauze electrode	Self-manufactured (Jinan, China).

**Table 3:** Analytic reagents used in the experiment.

Reagent Name	Chemical Formula	Molecular Weight	Purity	Manufacturer
Sodium chloride	NaCl	58.44	Analytic reagent	Sinopharm Chemical Reagent Co., Ltd. (Shanghai, China).
Potassium chloride	KCl	74.55	Analytic reagent	Sinopharm Chemical Reagent Co., Ltd. (Shanghai, China).
Anhydrous calcium chloride	CaCl_2_	110.99	Analytic reagent	Shantou Xilong Chemical Factory (Shantou, China).
Sodium bicarbonate	NaHCO_3_	84.01	Analytic reagent	Shantou Xilong Chemical Factory (Shantou, China).
Magnesium chloride	MgCl_2_·6H_2_O	203.3	Analytic reagent	Tianjin Fuchen Chemical Reagent Factory (Tianjin, China).
Anhydrous magnesium sulfate	MgSO_4_	120.37	Analytic reagent	Tianjin Guangcheng Chemical Reagent Co., Ltd. (Tianjin, China).
Monopotassium phosphate	KH_2_PO_4_	136.09	Analytic reagent	Tianjin Hongyan Chemical Reagent Factory (Tianjin, China).
Glucose	C_6_H_6_O_6_·H_2_O	198.17	Analytic reagent	Sinopharm Chemical Reagent Co., Ltd. (Shanghai, China).
Disodium hydrogen phosphate	Na_2_HPO_4_·12H_2_O	358.14	Analytic reagent	Sinopharm Chemical Reagent Co., Ltd. (Shanghai, China).
Deionized water	H_2_O	18	-	Chengdu Chaochun Science and Technology, Ltd. (Chengdu, China).

**Table 4:** Dependence of electrochemical corrosion properties of TA2 electrode on glucose concentration.

Concentration/mmol·L^−1^	5	7	10	12	15	20
*R*_s_ Ω	14.21	0.0001	0.0002531	3.299 × 10^−6^	70.33	9.986 × 10^−5^
*CPE*_e_	2.795 × 10^−5^	3.037 × 10^−5^	8.751 × 10^−7^	3.272 × 10^−5^	0.02373	5.904 × 10^−7^
*n*_e_	0.9199	0.8691	0.6803	0.8748	0.7534	0.7168
*R*_p1_/Ω	742.5	33.95	45.51	37	48.92	25.56
*CPE*_d_	1.004 × 10^−4^	2.343 × 10^−5^	3.17 × 10^−5^	1.778 × 10^−6^	3.388 × 10^−5^	3.159 × 10^−5^
*n*_d_	0.948	0.4093	0.8733	0.6563	0.8531	0.8748
*R*_p2_/Ω	6.057 × 10^5^	7.238 × 10^5^	7.439 × 10^5^	6.312 × 10^5^	8.308 × 10^5^	6.095 × 10^5^

**Table 5:** Electrochemical corrosion parameters determined by Tafel curves.

Glucose Concentration/mmol·L^−1^	*β_a_*/V^−1^	*β_c_*/V^−1^	*R*_p_/Ω	*E*_corr_/V	*I*_corr_/A
5	3.500	9.304	179,383	−0.459	1.934 × 10^−7^
7	3.045	13.787	123,767	−0.410	2.087 × 10^−7^
10	2.777	19.580	113,090	−0.372	1.72 × 10^−7^
12	3.007	13.824	120,237	−0.405	2.149 × 10^−7^
15	3.307	10.527	147,625	−0.442	2.129 × 10^−7^
20	3.427	9.328	155,732	−0.445	2.189 × 10^−7^
